# A Case of Carcinosarcoma of the Breast Presenting as Inflammatory Carcinoma and Review of the Literature

**DOI:** 10.7759/cureus.10104

**Published:** 2020-08-28

**Authors:** Harish Neelamraju Lakshmi, Devendra Saini, Prabha Om, Naveen Verma

**Affiliations:** 1 General Surgery, Sawai Man Singh Medical College, Jaipur, IND

**Keywords:** carcinosarcoma, metaplastic, biphasic, triple negative

## Abstract

Carcinosarcoma, also known as metaplastic carcinoma, is a rare and aggressive malignant tumor. We report a case of metaplastic carcinoma presenting as inflammatory carcinoma and provide a review of the related literature. A 38-year-old breastfeeding woman presented with concerns about a painful lump in her left breast. The symptoms had been present for two months. After admission to the hospital, the triple assessment revealed findings consistent with inflammatory carcinoma of the breast. The patient underwent modified radical mastectomy. Histopathological examination revealed a gray-white tumor with a biphasic pattern with features of ductal carcinoma as well as squamous and sarcomatous differentiation. On immunohistochemistry, the neoplastic cells were positive for cytokeratin and vimentin, and focally positive for smooth muscle antigen (SMA) and negative for estrogen receptor (ER), progesterone receptor (PR), and human epidermal growth factor receptor (HER-2/neu). Based on histological and immunohistochemical findings, the tumor was diagnosed as carcinosarcoma. Four of eighteen dissected axillary lymph nodes were positive for metastasis. Carcinosarcoma is often a triple-negative tumor. The lack of standardized treatment protocols frequently leads to poor prognosis and can pose a diagnostic dilemma; it should be part of the differential diagnosis for a case of carcinoma of the breast presenting as inflammatory carcinoma.

## Introduction

Carcinosarcoma is a rare and aggressive malignant tumor, accounting for <0.1% of all breast malignancies [1]. Also known as metaplastic carcinoma, it was first described by Huvos et al. in 1973 as a rare form of mammary cancer with mixed epithelial and sarcomatoid components [2]. We report a case of a metaplastic carcinoma presenting as inflammatory carcinoma and describe its clinical, pathological, and immunohistochemical features in the context of a review of the literature.

## Case presentation

A 38-year-old breastfeeding married woman with three children presented with a two-month history of a rapidly growing painful lump in her left breast. The patient described the pain as burning. There was no significant past medical or surgical history, and her family history was negative for any malignancy in first degree relatives. The patient had a normal menstrual history (age at menarche: 14 years).

**Figure 1:**
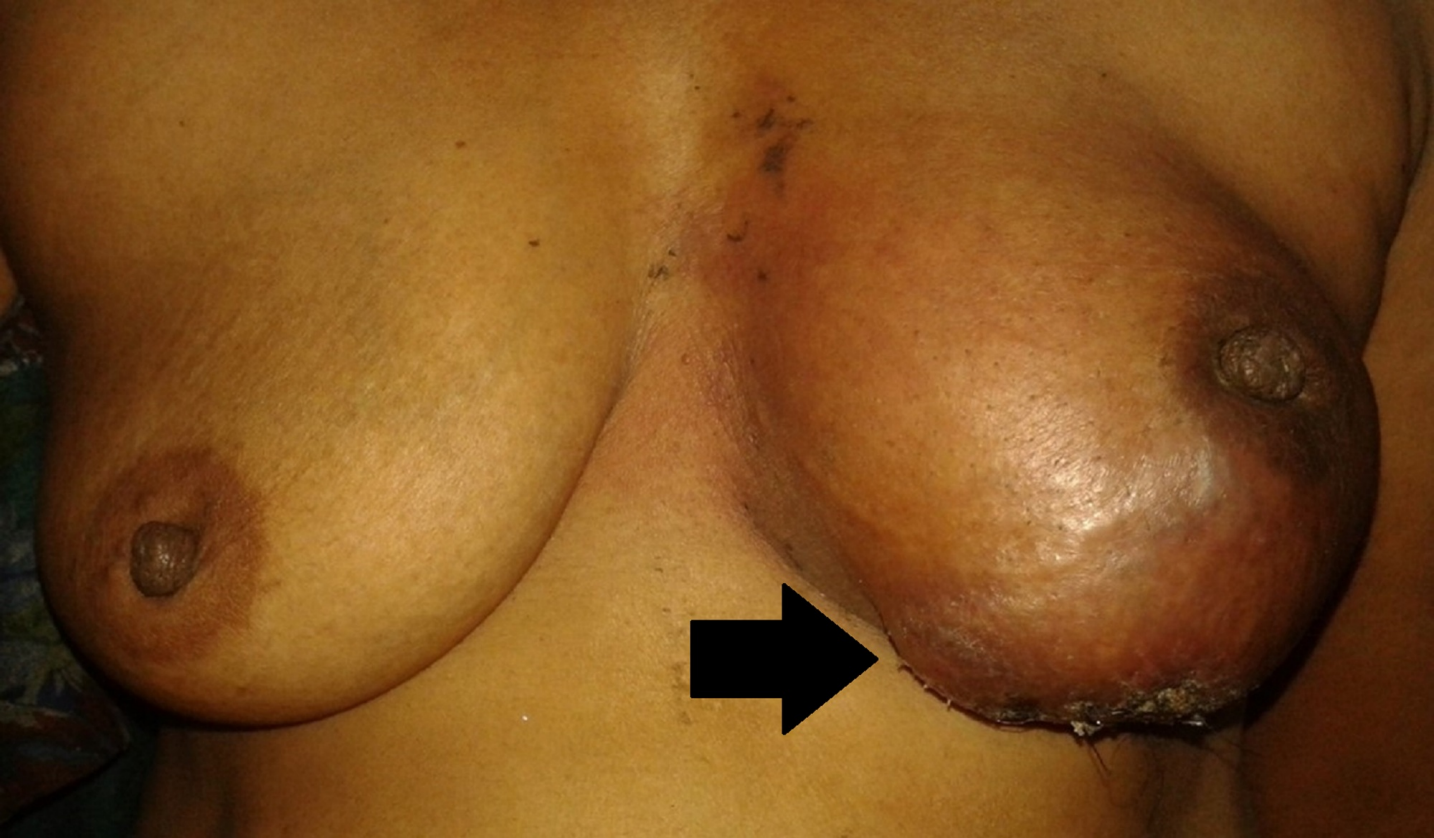
Clinical presentation of the patient

On examination, the left breast was enlarged and erythematous, with dimpling of the overlying skin and nipple retraction. Palpation revealed a 7 x 6 cm warm and tender lump in the lower half of the left breast. The lesion was irregularly shaped and had variable consistency (Figure 1). The left anterior axillary lymph nodes were enlarged, mobile, and firm, with the largest node measuring 2 x 1 cm.

**Figure 2:**
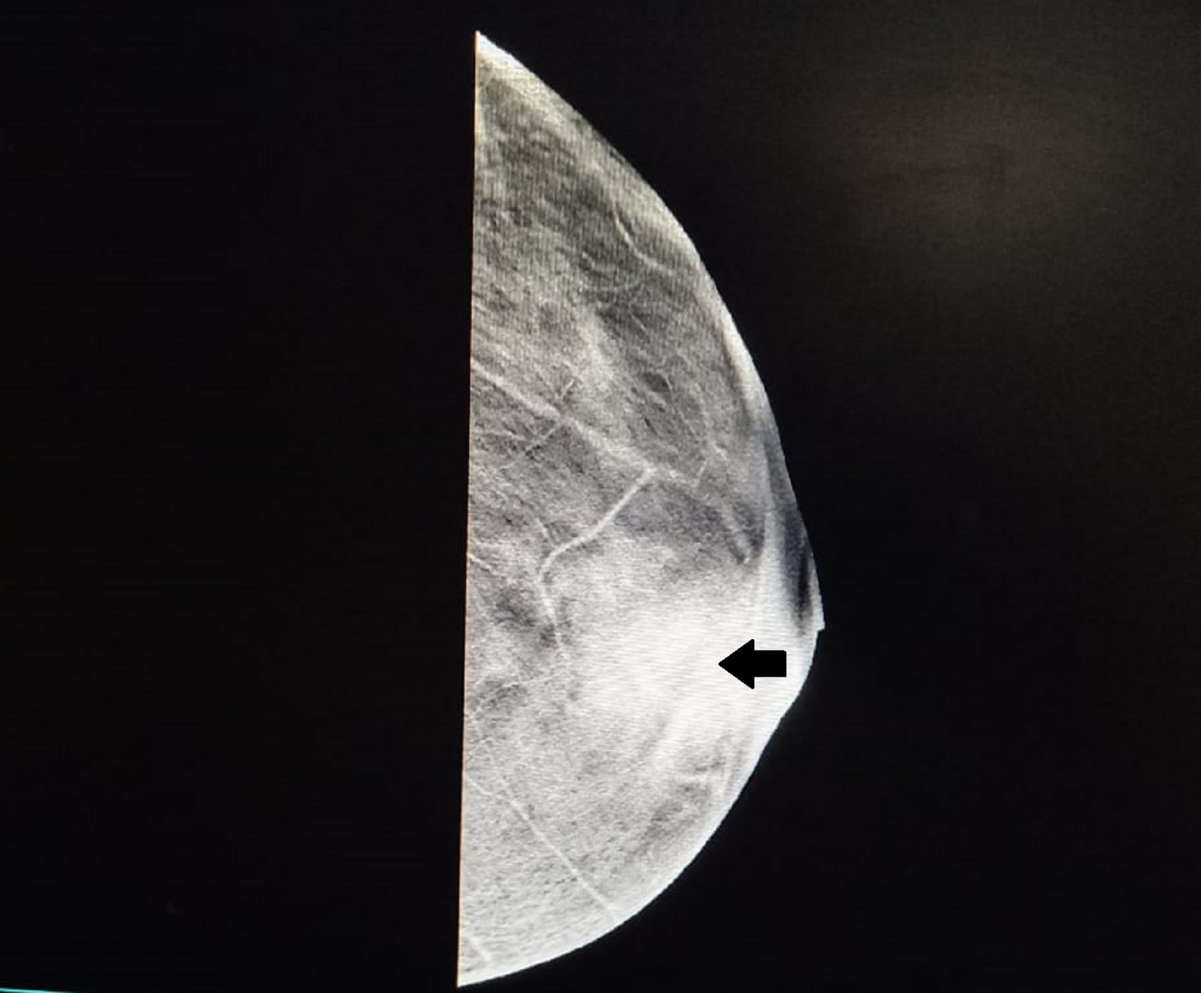
Mammography showing lesion in the lower half of the breast

Routine laboratory testing revealed leukocytosis (total leukocyte count = 12,950 cells/cu.mm). Ultrasound imaging of the breast showed a large hypoechoic lesion with spiculated margins, increased vascularity, and calcification, in addition to multiple enlarged axillary lymph nodes. Mammography revealed a mixed radio-opaque lesion in the lower inner and outer quadrants of the left breast suggestive of a grade IV Breast Imaging Reporting and Data System lesion (Figure 2).

**Figure 3:**
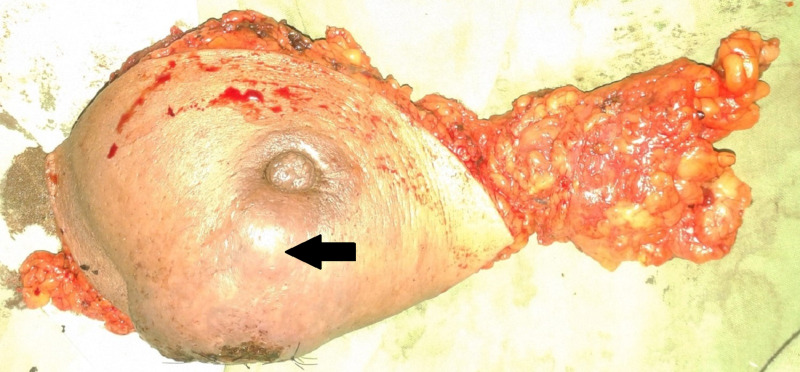
Left modified radical mastectomy specimen

Core needle biopsy revealed highly pleomorphic malignant cells arranged in nests, fascicles, and irregular storiform pattern with mitosis 30/10 high-power field. The differential diagnosis included carcinosarcoma and high-grade sarcoma. The patient underwent modified radical mastectomy with an uneventful postoperative course (Figure 3). 

**Figure 4:**
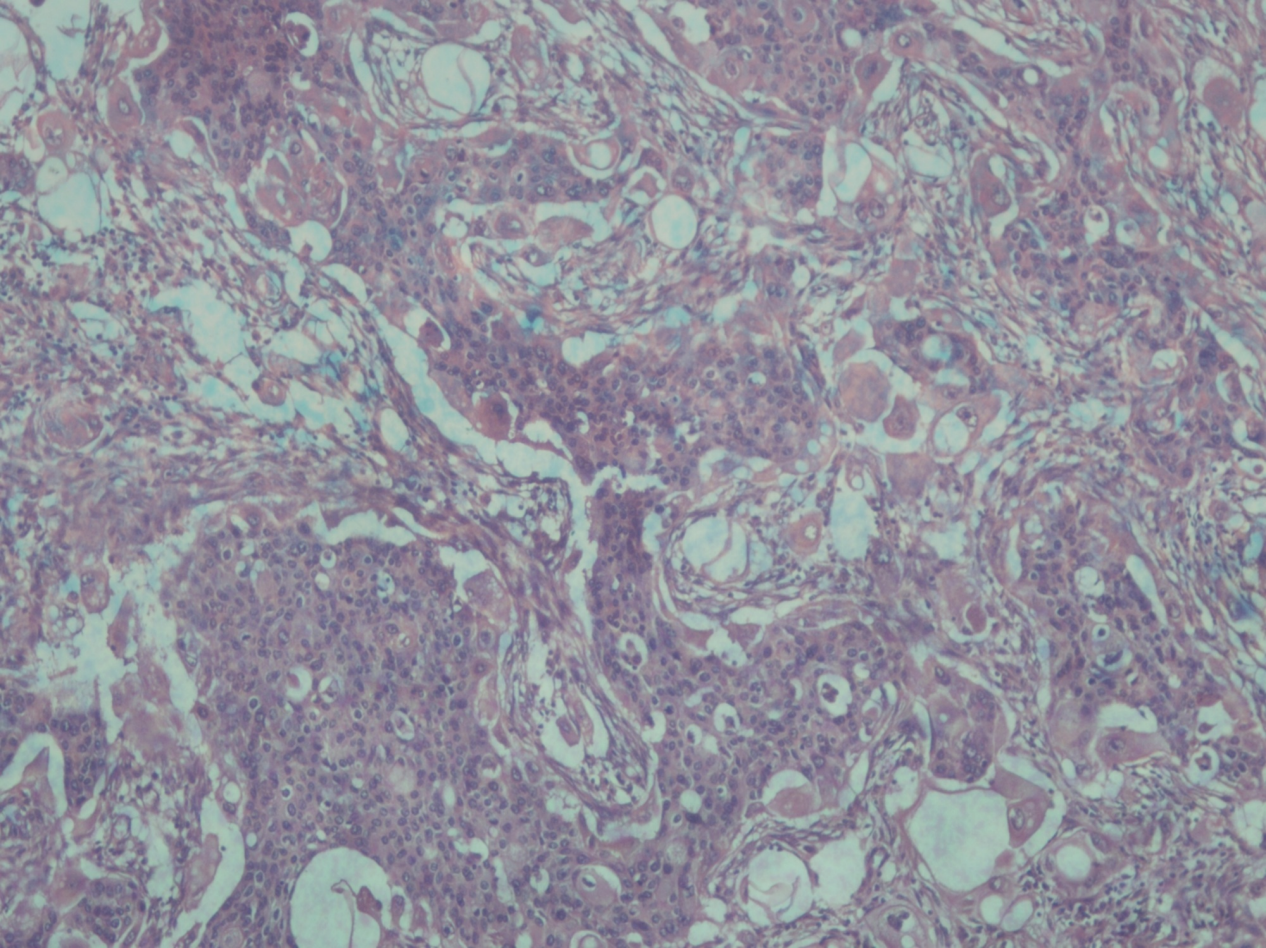
Histopathologic image of carcinosarcoma

**Figure 5:**
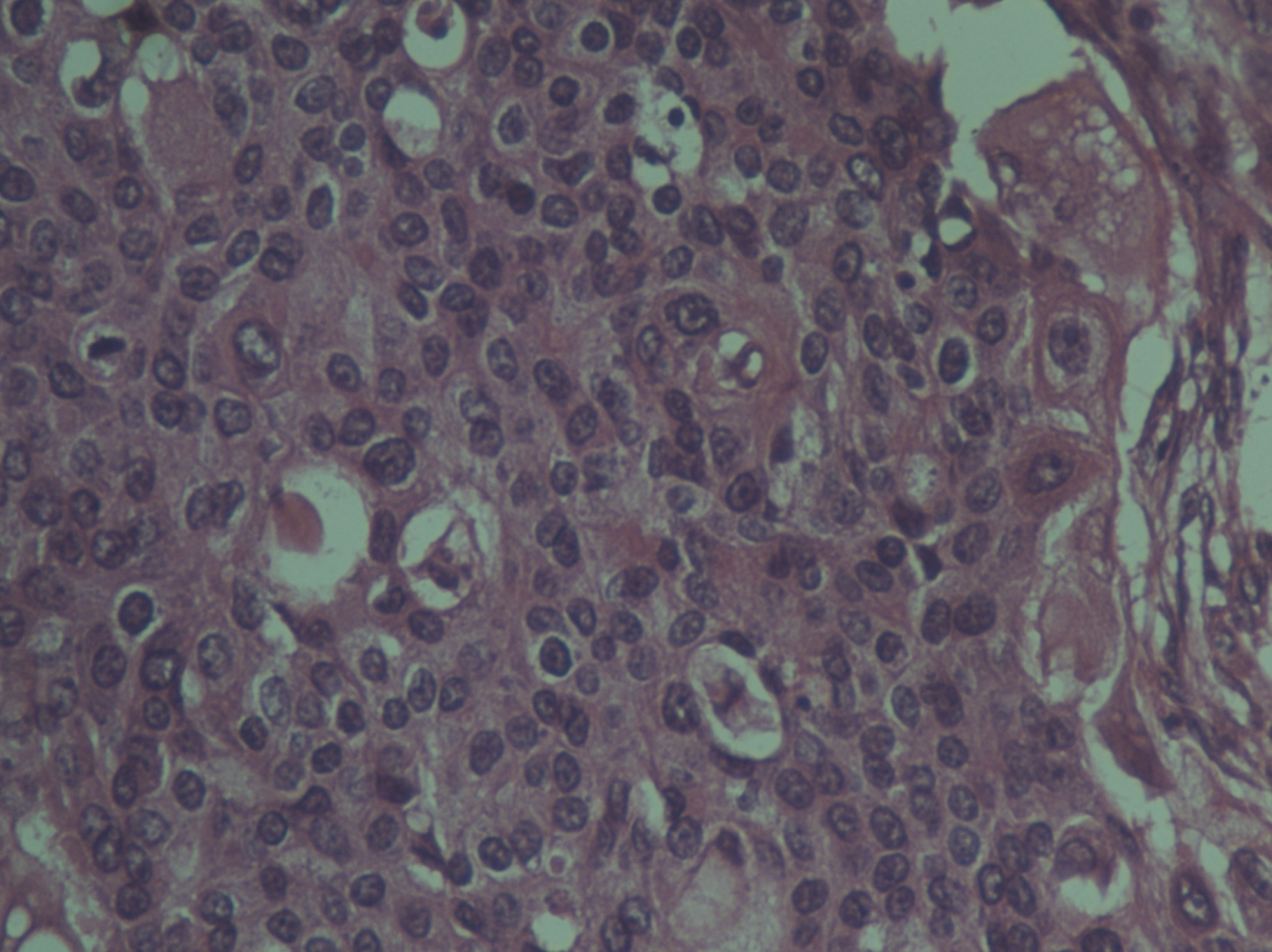
Histopathologic image with ×40 magnification

**Figure 6:**
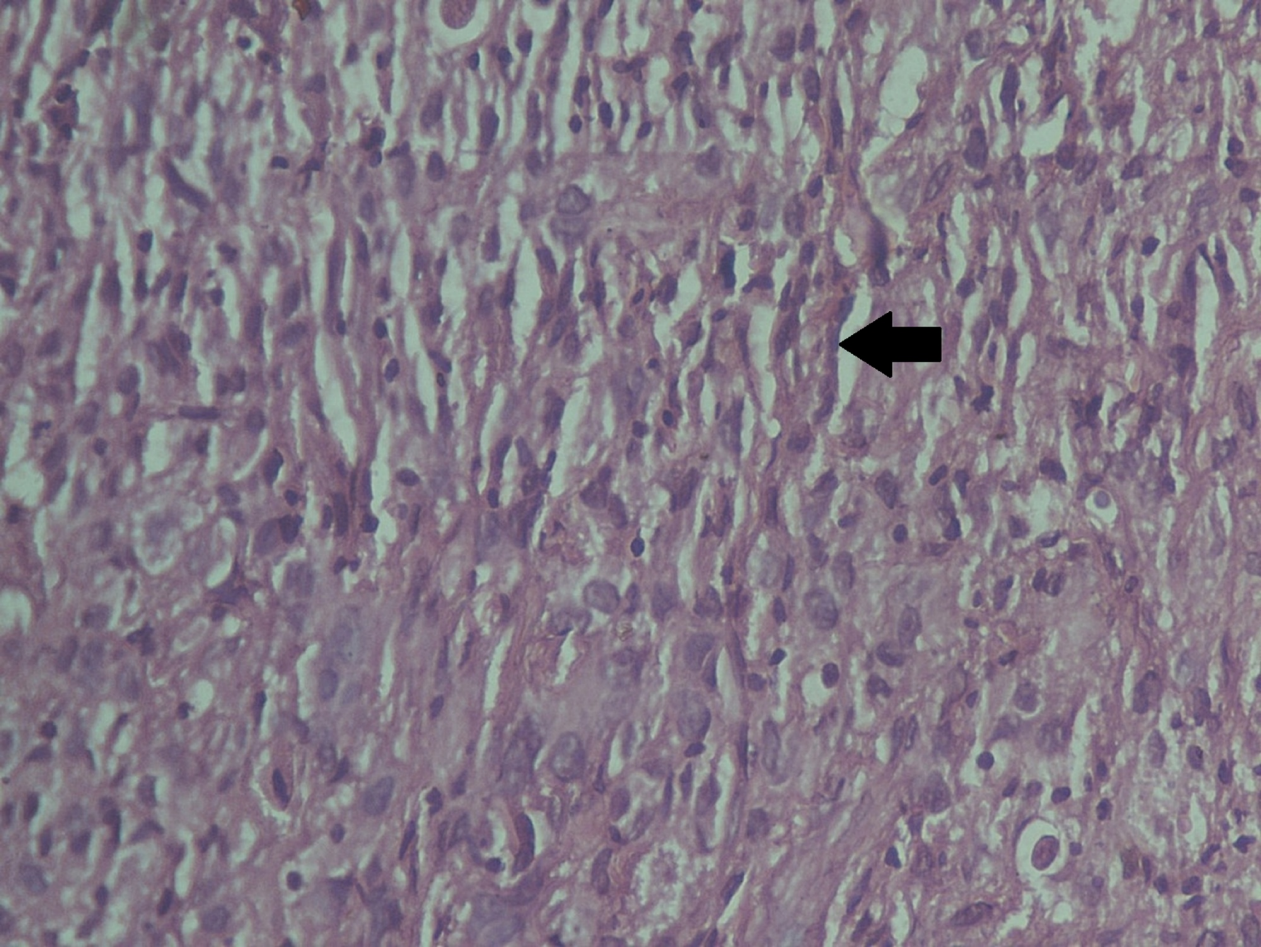
Histopathology showing sarcomatous differentiation

**Figure 7:**
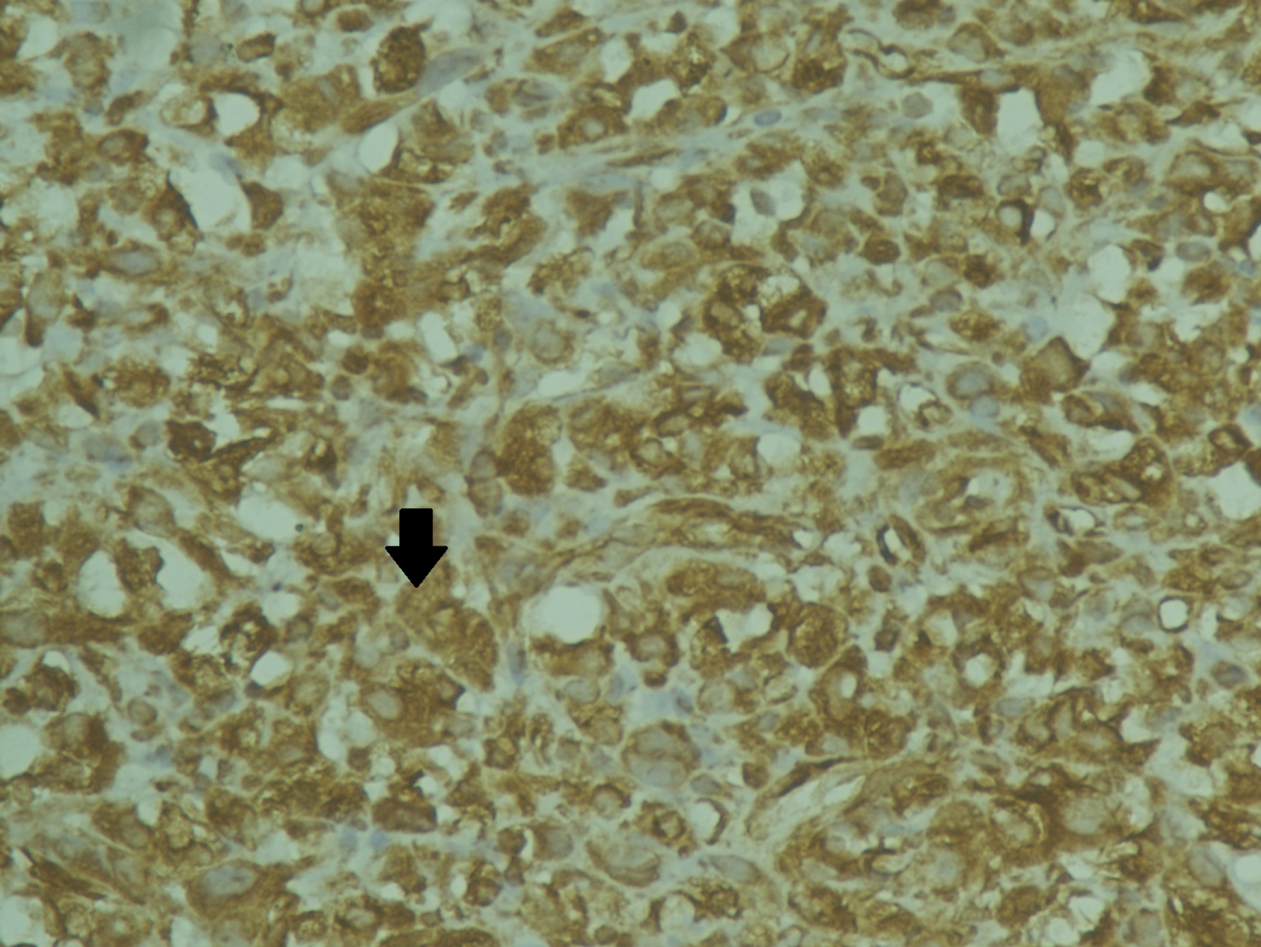
Immunohistochemical staining positive for vimentin

**Figure 8:**
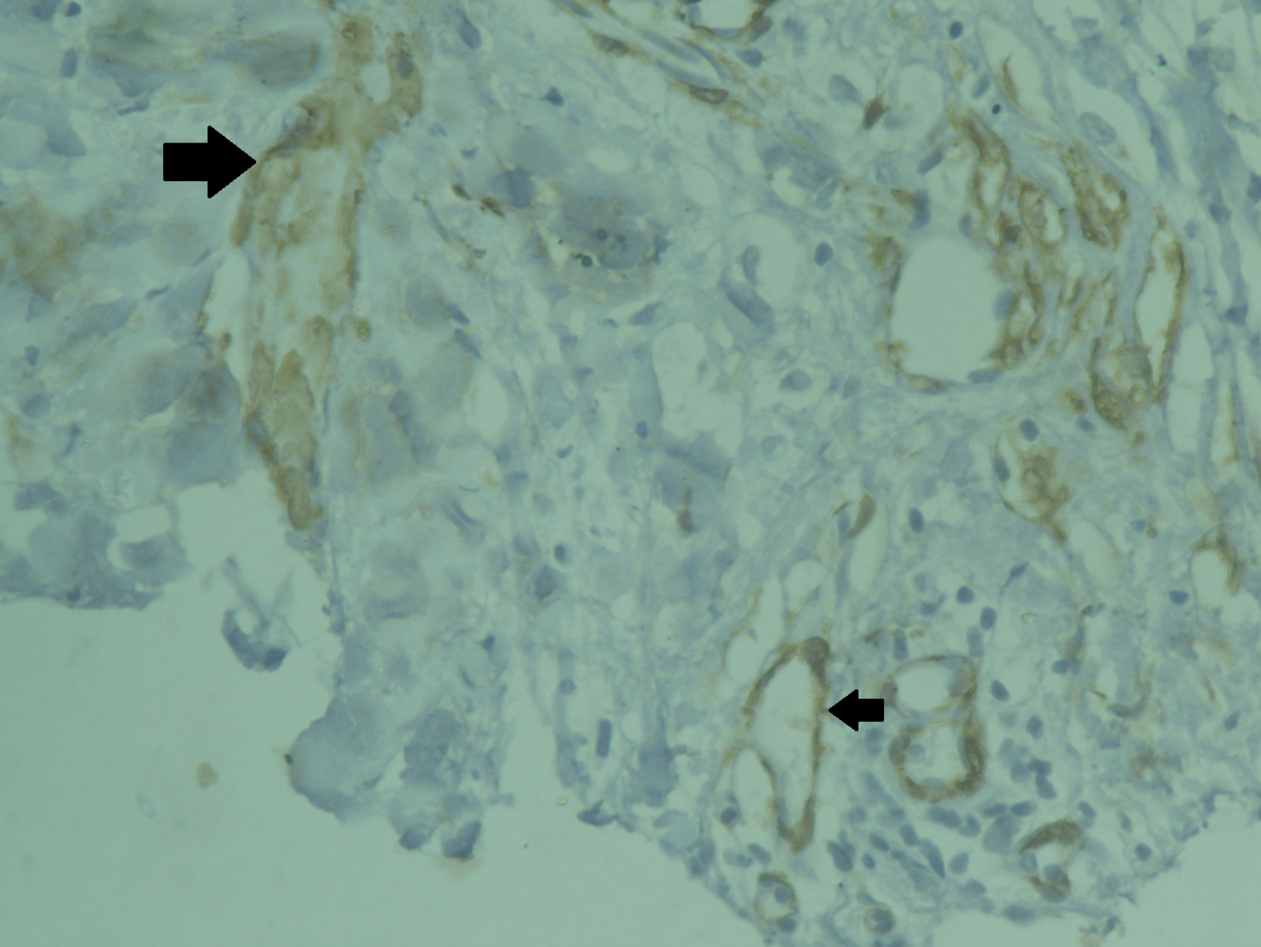
Immunohistochemical staining positive for smooth muscle antigen

The histopathological analysis reported a 6 x 6 x 6 cm gray-white tumor with biphasic pattern with features of ductal carcinoma as well as squamous and sarcomatous differentiation (Figures 4-8). On immunohistochemistry, the neoplastic cells were positive for cytokeratin and vimentin, focally positive for smooth muscle antigen (SMA), and negative for estrogen receptor (ER), progesterone receptor (PR), and human epidermal growth factor receptor (HER-2/neu). Overall, features were in favor of a diagnosis of metaplastic carcinoma or carcinosarcoma. 

## Discussion

Carcinosarcoma is an extremely rare neoplasm that occasionally occurs in organs like the ovary or uterus. The neoplasm is characterized by a biphasic pattern (carcinomatous and mesenchymal) without a transition zone in between [3]. Although controversial, the consensus is that the tumor originates from myoepithelial cells through a phenotypic transformation of epithelial cells and can arise from a pre-existing fibroadenoma or cystosarcoma phyllodes [4-6]. A totipotent cell with biphasic differentiation has been linked to the development of metaplastic carcinoma [7]. The neoplasm is an admixture of two or more components, including adenosquamous, adenocarcinoma, undifferentiated, matrix, spindle cell, fibroblastic, chondroblastic, osteoblastic, or sarcomatous elements.

The World Health Organization’s histological classification for tumors of the breast classifies metaplastic carcinoma into pure epithelial metaplastic carcinomas, squamous cell carcinoma, adenocarcinoma with spindle cell metaplasia, adenosquamous carcinoma, mucoepidermoid carcinoma, or mixed epithelial/mesenchymal metaplastic carcinomas. The mean age at diagnosis lies between the fourth and fifth decades of life (i.e., the perimenopausal age group) [8,9]. The median tumor size is reported to range from 3.4 to 5.7 cm in various series [10-12]. Axillary node involvement is usually less frequent in comparison to invasive ductal carcinoma (IDC), although it differs across case series. The neoplasm metastasizes by hematogenous spread to the lungs, pleura, liver, skeletal system, or brain.

Modified radical mastectomy is the preferred surgical procedure as a large tumor size is a contraindication for breast-conserving surgery. Carcinosarcomas are poorly differentiated aggressive neoplasms that often tend to be triple-negative (ER, PR, and HER-2/neu). Adjuvant chemoradiotherapy is necessary for locoregional control. Hormonal therapy is ineffective as these tumors are usually triple negative. Anthracycline-based chemotherapy is more effective than cyclophosphamide methotrexate fluorouracil regimen [13,14]. Adjuvant radiotherapy has been shown to decrease the risk of death by 33% in mastectomy patients [15]. Overexpression of Her1/epidermal growth factor receptor (EGFR) suggests that agents like gefitinib and cetuximab, which target the EGFR, may play a role in the treatment of metaplastic carcinoma [16,17]. Current National Comprehensive Cancer Network guidelines for invasive breast cancer suggest similar management for metaplastic carcinoma as for IDC.

## Conclusions

This case illustrates a rare aggressive variant of carcinoma breast, metaplastic breast cancer, the chimera among breast neoplasms which differs from IDC in various aspects. The development of standardized treatment protocols is needed for expert management. Physicians should be aware of this important differential diagnosis for inflammatory carcinoma breast.

## References

[1] Abbasi MA, Mahmood H, Faheem M, Khan KA, Irfan J (2012). Carcinosarcoma of the breast. J Coll Physicians Surg Pak.

[2] Huvos AG, Lucas JC Jr, Foote FW Jr (1973). Metaplastic breast carcinoma: rare form of mammary cancer. N Y State J Med.

[3] Wargotz ES, Norris HJ (1989). Metaplastic carcinomas of the breast. III. Carcinosarcoma. Cancer.

[4] Harris M, Persaud V (1974). Carcinosarcoma of the breast. J Pathol.

[5] Bolton B, Sieunarine K (1990). Carcinosarcoma: a rare tumour of the breast. Aust N Z J Surg.

[6] Teixeira MR, Qvist H, Bohler PJ, Pandis N, Heim S (1998). Cytogenetic analysis shows that carcinosarcomas of the breast are of monoclonal origin. Genes Chromosomes Cancer.

[7] Wada H, Enomoto T, Tsujimoto M, Nomura T, Murata Y, Shroyer KR (1998). Carcinosarcoma of the breast: molecular-biological study for analysis of histogenesis. Hum Pathol.

[8] Yakan S, Sarı E, Erkan N (2014). Breast carcinosarcomas. J Breast Health.

[9] Altaf FJ, Mokhtar GA, Emam E, Bokhary RY, Mahfouz NB, Al Amoudi S, AL-Gaithy ZK (2014). Metaplastic carcinoma of the breast: an immunohistochemical study. Diagn Pathol.

[10] Samoon Z, Beg M, Idress R, Jabbar AA (2019). Survival and treatment outcomes of metaplastic breast carcinoma: Single tertiary care center experience in Pakistan. Indian J Cancer.

[11] Esbah O, Turkoz FP, Turker I (2012). Metaplastic breast carcinoma: case series and review of the literature. Asian Pac J Cancer Prev.

[12] Koual M, Cano-Sancho G, Bats AS (2019). Associations between persistent organic pollutants and risk of breast cancer metastasis. Environ Int.

[13] Tokudome N, Sakamoto G, Sakai T (2005). A case of carcinosarcoma of the breast. Breast Cancer.

[14] Hennessy BT, Giordano S, Broglio K (2006). Biphasic metaplastic sarcomatoid carcinoma of the breast. Ann Oncol.

[15] Tseng WH, Martinez SR (2011). Metaplastic breast cancer: to radiate or not to radiate?. Ann Surg Oncol.

[16] Beatty JD, Atwood M, Tickman R, Reiner M (2006). Metaplastic breast cancer: clinical significance. Am J Surg.

[17] Okubo S, Kurebayashi J, Otsuki T, Yamamoto Y, Tanaka K, Sonoo H (2004). Additive antitumour effect of the epidermal growth factor receptor tyrosine kinase inhibitor gefitinib (Iressa, ZD1839) and the antioestrogen fulvestrant (Faslodex, ICI 182,780) in breast cancer cells. Br J Cancer.

